# Spectroscopic photoacoustic imaging of cervical tissue composition in excised human samples

**DOI:** 10.1371/journal.pone.0247385

**Published:** 2021-03-03

**Authors:** Yan Yan, Maryam Basij, Alpana Garg, Aneesha Varrey, Ali Alhousseini, Richard Hsu, Edgar Hernandez-Andrade, Roberto Romero, Sonia S. Hassan, Mohammad Mehrmohammadi

**Affiliations:** 1 Department of Biomedical Engineering, Wayne State University College of Engineering, Detroit, Michigan, United States of America; 2 Department of Internal Medicine, Wayne State University School of Medicine, Detroit, Michigan, United States of America; 3 Division of Obstetrics and Maternal-Fetal Medicine, Division of Intramural Research, Perinatology Research Branch, Eunice Kennedy Shriver National Institute of Child Health and Human Development, National Institutes of Health, U.S. Department of Health and Human Services, Bethesda, Maryland and Detroit, Michigan, United States of America; 4 Department of Obstetrics and Gynecology, Wayne State University School of Medicine, Detroit, Michigan, United States of America; 5 Department of Physiology, Wayne State University School of Medicine, Detroit, Michigan, United States of America; 6 Department of Obstetrics and Gynecology, William Beaumont Hospital, Royal Oak, Michigan, United States of America; 7 Department of Obstetrics and Gynecology and Reproductive Sciences, McGovern Medical School, University of Texas, Health Science Center at Houston (UTHealth), Houston, Texas, United States of America; 8 Department of Obstetrics and Gynecology, University of Michigan, Ann Arbor, Michigan, United States of America; 9 Department of Epidemiology and Biostatistics, Michigan State University, East Lansing, Michigan, United States of America; 10 Center for Molecular Medicine and Genetics, Wayne State University, Detroit, Michigan, United States of America; 11 Detroit Medical Center, Detroit, Michigan, United States of America; 12 Department of Obstetrics and Gynecology, Florida International University, Miami, Florida, United States of America; 13 Office of Women’s Health, Wayne State University School of Medicine, Detroit, Michigan, United States of America; 14 Department of Electrical and Computer Engineering, Wayne State University, Detroit, Michigan, United States of America; 15 Barbara Ann Karmanos Cancer Institute, Detroit, Michigan, United States of America; University of Insubria, ITALY

## Abstract

**Objective:**

Cervical remodeling is an important component in determining the pathway of parturition; therefore, assessing changes in cervical tissue composition may provide information about the cervix’s status beyond the measurement of cervical length. Photoacoustic imaging is a non-invasive ultrasound-based technology that captures acoustic signals emitted by tissue components in response to laser pulses. This optical information allows for the determination of the collagen-to-water ratio (CWR). The purpose of this study was to compare the CWR evaluated by using spectroscopic photoacoustic (sPA) imaging in cervical samples obtained from pregnant and non-pregnant women.

**Methods:**

This cross-sectional study comprised cervical biopsies obtained at the time of hysterectomy (n = 8) and at the scheduled cesarean delivery in pregnant women at term who were not in labor (n = 8). The cervical CWR was analyzed using a fiber-optic light-delivery system integrated to an ultrasound probe. The photoacoustic signals were acquired within the range of wavelengths that cover the peak absorption of collagen and water. Differences in the CWR between cervical samples from pregnant and non-pregnant women were analyzed. Hematoxylin and eosin and Sirius Red stains were used to compare the collagen content of cervical samples in these two groups.

**Results:**

Eight cervix samples were obtained after hysterectomy, four from women ≤41 years of age and four from women ≥43 years of age; all cervical samples (n = 8) from pregnant women were obtained after 37 weeks of gestation at the time of cesarean section. The average CWR in cervical tissue samples from pregnant women was 18.7% (SD 7.5%), while in samples from non-pregnant women, it was 55.0% (SD 20.3%). There was a significantly higher CWR in the non-pregnant group compared to the pregnant group with a p-value <0.001. A subgroup analysis that compared the CWR in cervical samples from pregnant women and non-pregnant women ≤41 years of age (mean 46.3%, SD 23.1%) also showed a significantly higher CWR (p <0.01). Lower collagen content in the pregnancy group was confirmed by histological analysis, which revealed the loss of tissue composition, increased water content, and collagen degradation.

**Conclusion:**

The proposed bimodal ultrasound and sPA imaging system can provide information on the biochemical composition of cervical tissue in pregnant and non-pregnant women. Photoacoustic imaging showed a higher collagen content in cervical samples from non-pregnant women as compared to those from pregnant women, which matched with the histological analysis. This novel imaging method envisions a new potential for a sensitive diagnostic tool in the evaluation of cervical tissue composition.

## Introduction

The human cervix is a dynamic structure [1–3] comprising smooth muscle, fibroblasts, epithelial tissue, and blood vessels stabilized within a network of fibrous connective tissue composed of 70% type I collagen and 30% type III collagen as well as elastin and proteoglycans [4–6]. Throughout pregnancy, multiple phases of remodeling and reorganization result in changes of cervical “consistency” (stiffness, elasticity) and length [7]. These changes occur during the reorganization of the collagen network in cervical tissue [8–12], which increases progressively and at a higher rate closer to delivery [13,14]. The shortening of the cervical length before the full term of gestation has been associated with a higher risk of preterm delivery.

Cervical remodeling occurs throughout gestation and is characterized by the reorganization of collagen structure, increased hydration, degradation of extracellular matrix proteins, and increased vascularity that alter the cervix’s consistency and length [15–18]. Evaluating these biochemical changes in conjunction with cervical length may help increase the prediction of preterm birth [19–23]. Previous studies demonstrated that preterm birth occurs when structural remodeling or disorganization of the cervical collagen/muscular network begins earlier than expected [24,25].

Transvaginal ultrasound (TVUS) measurement of the cervical length is currently the main imaging technique used to evaluate changes in the cervix during pregnancy [26,27]. Alternative ultrasound (US) techniques, such as elastography [28,29] and attenuation, provide quantitative measures of structural and biomechanical properties of cervical tissue. However, these modalities garner no information about the underlying mechanisms of cervical tissue remodeling, which is required to more accurately determine the timing and success of labor induction. During the past decade, several non-invasive optical techniques have been described to evaluate the collagen network remodeling. These include Raman spectroscopy [30], light-induced fluorescence [31], second harmonic generation (SHG) [32], infra-red spectroscopy [33], and optical coherence tomography (OCT) [34,35]. However, these techniques are mostly optical with a very narrow region of interest and need a separate probe from the transvaginal ultrasound (TVUS) transducer. Furthermore, the quantitative data is obtained without an ultrasound image, which is difficult to implement in clinical settings (OCT and SHG). Therefore, novel imaging instrumentation and methods that allow for quantitative visualization of molecular and microstructural changes in the cervical stroma (i.e., collagen fibers, microvasculature) and smooth muscle cell networks may provide objective, quantitative data on the process of cervical remodeling and might contribute to the determination of the optimal time for induction of labor.

Photoacoustic (PA) imaging has been applied for the evaluation of different organs [36–40], such as the brain [41,42], liver [43], cervix [44–48], lymph nodes [49], aorta [50], and breast [51], to quantify biochemical constituents and to aid in cancer diagnosis and management, such as skin [52], cervical [53], and breast cancers [54–57]. Given its ability to acquire functional and molecular images with a high resolution at clinically relevant depths, in addition to sharing common signal-acquisition hardware with ultrasound, PA imaging can serve as a valuable adjunct to cervical length measurement [46,58] by providing additional information on the cervix’s composition. Our objective was to perform PA profiling of the collagen-to-water ratio (CWR) in *ex-vivo* cervix samples from pregnant and non-pregnant women to validate its capability during cervical remodeling in pregnancy.

## Materials and methods

This cross-sectional study was conducted at the Labor & Delivery Research Unit and the Gynecology Unit of the Detroit Medical Center/Hutzel Women’s Hospital and the Perinatology Research Branch, *Eunice Kennedy Shriver* National Institute of Child Health and Human Development (NICHD), National Institutes of Health, U.S. Department of Health and Human Services, Wayne State University School of Medicine, and the Department of Biomedical Engineering, Wayne State University College of Engineering, Detroit, Michigan. All patients provided written informed consent prior to the collection of the cervical biopsies and were enrolled in research protocol (040302M1F) approved by the Human Investigation Committee of Wayne State University and the Institutional Review Board of NICHD. Eight women undergoing total hysterectomy due to uterine bleeding or premalignant lesions, and eight women with a singleton term pregnancy without labor scheduled for an elective cesarean section, were included in the current study. Demographic data and clinical characteristics of all patients were obtained and registered.

### Cervix sample collection and preparation

The cervical samples were divided into two groups, with eight samples per group, collected after hysterectomy and during cesarean delivery procedures, respectively. For the hysterectomy group, the cervical samples were taken immediately after the removal of the uterus in the operating room. For each individual, a five-cubic-millimeter (mm^3^) cervical sample was excised from the 12 o’clock position of the cervix. The excised sample was rinsed with phosphate-buffered saline (PBS, Corning^®^, 1X without calcium and magnesium, PH 7.4 ± 0.1) to remove blood from the tissue surface before placement into Ambion^®^ RNALater^™^ (Invitrogen Inc.) storage solution at 4°C. During imaging and transportation, samples were kept in RNALater at 4°C. After imaging, samples were embedded into the O.C.T. compound (Tissue-Tec, Sakura Finetek Inc.) and frozen with liquid nitrogen. Several (>2) ten-micrometer-thick sections were sliced using a cryostat and separated into two groups. Samples from one group were stained with hematoxylin and eosin (H&E), and samples from the other group were stained with Sirius Red for further histological analyses.

Cervical samples from women who underwent cesarean delivery were taken from the 12 o’clock position of the cervix after the procedure. Under aseptic conditions, a sterile speculum was placed, and Kevorkian biopsy forceps were used to obtain the cervical sample. The sample was rinsed with PBS to remove blood on the tissue surface before preservation in RNALater. Imaging, transportation, and histology procedures followed the same protocol as the cervix-hysterectomy samples.

### Principles of photoacoustic imaging to measure collagen-to-water ratio (CWR)

The proposed bimodal imaging system includes a TVUS probe coupled to a fiber-optic light delivery system surrounding the TVUS transducer for spectroscopic photoacoustic (sPA) imaging. This custom-built light delivery system consists of 19 fibers (NA = 0.39, Thorlabs, Inc., USA). Of the 19 fibers, 18 delivered light around the TVUS probe, and one fiber was used to monitor real-time energy. The fiber-holding sheath is designed to hold the fibers in place. The total diameter of this combined probe is 29 mm, which fits well with clinical requirements. The fibers were bent close to the probe’s tip to enhance the illumination patterns. A Monte-Carlo model simulation to optimize the alignment and bending angles of the fibers was performed to achieve the optimal light delivery for PA imaging [59]. The proximal end of the fiber bundle was coupled to a self-cooled nanosecond, tunable pulsed laser (Phocus Core, OPOTek^®^ Inc., USA) across a range of wavelengths between 680 nm to 2500 nm at a pulse repetition of 10 Hz. A programmable digital US acquisition system (Vantage 128, Verasonics^®^, Inc., USA) acquired US and PA images. A custom-built, high-speed, field-programmable gate array synchronized laser excitation and US acquisition and controlled the interleaved acquisition of US and PA frames. Interleaved US and PA images were reconstructed by using an adaptive beamforming algorithm [60]. Real-time TVUS and PA imaging sequences were developed in which three US frames were acquired between two consecutive PA frames, all synchronization signals were calibrated, and co-registered TVUS/PA images were reconstructed. Additionally, the imaging of a calibration phantom characterized the axial resolution of 260 μm and lateral resolution (at 25 mm depth) of 450 μm [44].

### Spectroscopic photoacoustic evaluation of CWR

**Fig 1:**
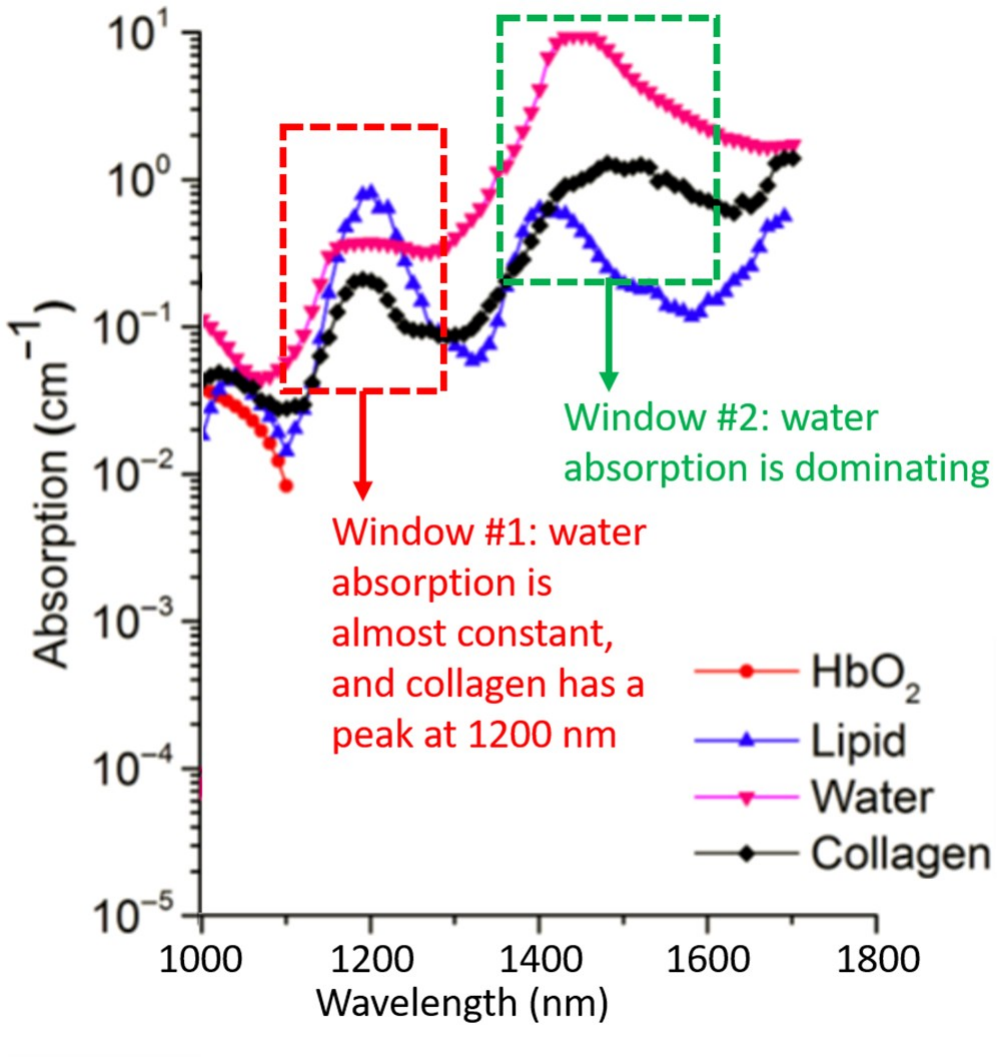
Optical absorptions of oxyhemoglobin, lipid, collagen, and water. In the collagen dominating (window #1), water has nearly constant absorption and, by contrast, collagen peaks around 1200 nm. In the water dominating (window #2), water peaks about 1470 nm, and collagen peaks around 1520 and 1540 nm (figure adopted from [61]).

Spectroscopic PA (sPA) is a widely used PA imaging technology that utilizes laser illumination at different wavelengths to the tissue of interest and probes the object’s optical properties as broadband acoustic pressure waves. To estimate the CWR in cervical tissue, we developed a two-step sPA-CWR measurement algorithm. First, amplification of the PA signal acquired between 1150 and 1250 nm (window 1: collagen domination, where collagen has its peak absorption) detected the relatively small variations [61]. At wavelength (λ) of 1150 to 1250 nm, water absorption remained constant, but collagen absorption had a clear peak [62,63]. Since collagen is still relatively weak compared to water (Fig 1) [61], we used an algorithm (Eq 1) [64] to amplify collagen’s PA.

Later, the amplified images were passed through a standard sPA wavelength unmixing method (Eq 2) [41] to calculate the final CWR. This spectroscopic process, performed for each pixel of the image, was guided by the B-mode US to identify the true location of the cervical tissue. The sPA wavelength unmixing method took the average of two pairs of PA data, which were 1150 with 1200 and 1200 with 1250. The higher wavelength features (window 2: water dominating) were not applied in the unmixing method given that water has a strong absorption peak at λ = ~1450 nm. The CWR calculation method may be further enhanced by including sPA data acquired between 1400 and 1600 nm.

$$PA_{Amplified}(\lambda )=\frac{\varepsilon_{\text{C}\lambda 1200}}{\varepsilon_{\text{C}\lambda 1150}}\cdot \frac{\varepsilon_{\text{C}\lambda 1200}}{\varepsilon_{\text{C}\lambda 1250}}\cdot PA_{\lambda }$$ (1)

$$\begin{array}{cc} CWR & =\frac{\left[Collagen_{PA}\right]}{\left[Collagen_{PA}]+[Water_{PA}\right]}\\ & =\frac{\varepsilon_{W_{\lambda }{}_{2}}\cdot PA_{\lambda_{1}}-\varepsilon_{W_{\lambda }{}_{1}}\cdot PA_{\lambda_{2}}}{PA_{\lambda_{1}}(\varepsilon_{W_{\lambda_{2}}}-\varepsilon_{C_{\lambda }{}_{2}})-PA_{\lambda_{2}}(\varepsilon_{W_{\lambda_{1}}}-\varepsilon_{C_{\lambda }{}_{1}})} \end{array}$$ (2)

where the *ε*_*λ*_ is the mass extinction coefficient for the absorbers (Collagen C, Water W) at the wavelength λ, and the *PA*_*λ*_ represents the received PA signal amplitude at wavelength λ.

### Collagen phantoms

**Fig 2:**
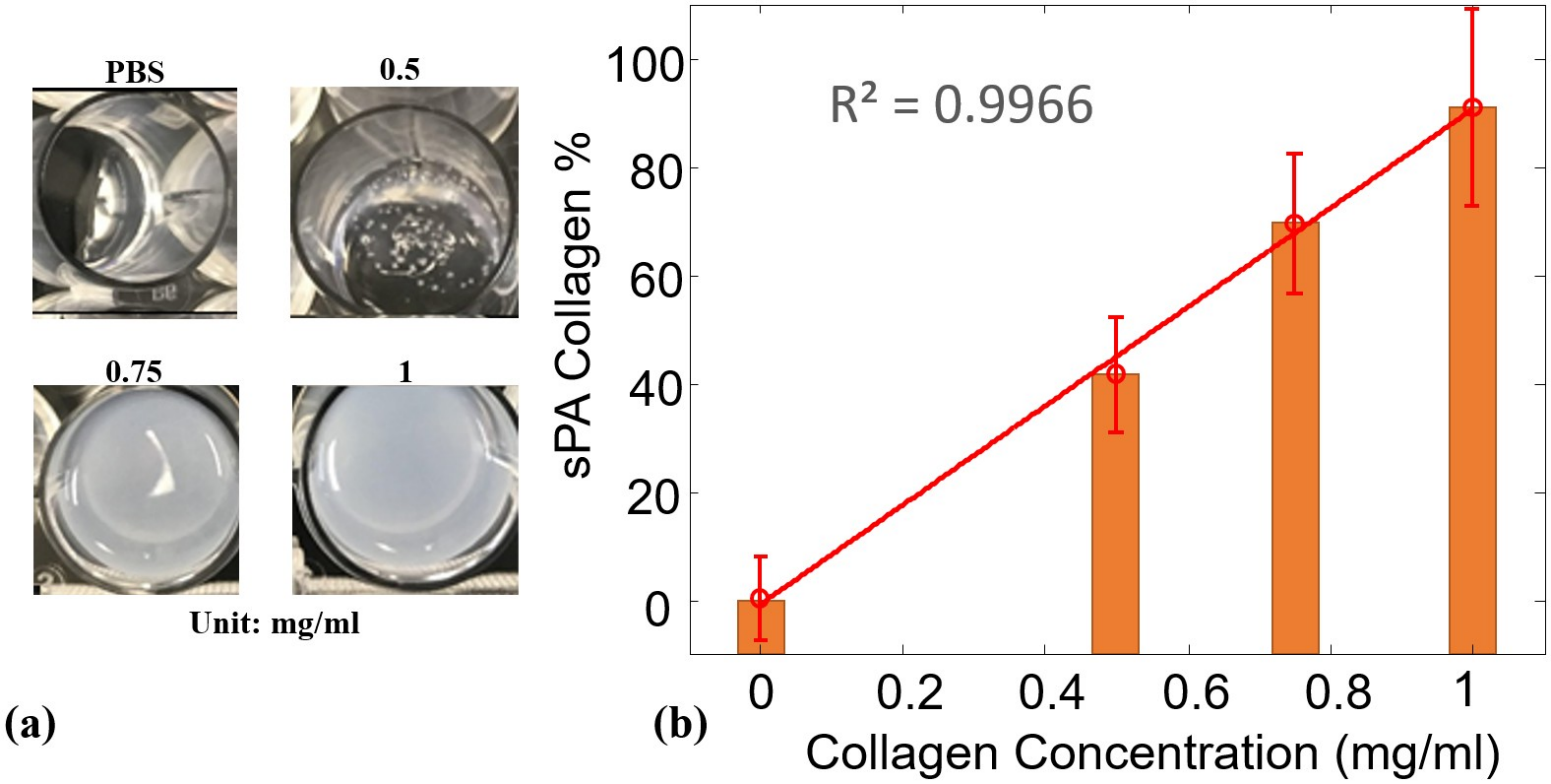
Collagen-to water-ratio in collagen phantoms. (a) Photograph of collagen phantoms at different concentrations ranging from 0 (PBS) to 1 mg/mL. (b) Normalized sPA-CWR to estimate the collagen concentration for the collagen phantoms at different concentrations. The linear curve fit showed a high correlation between the detected CWR to the collagen concentration, with the coefficient of determination (R2) of 99.66%.

Before applying the sPA-CWR method in human samples, we validated the method in collagen phantoms with known concentrations (1.0, 0.75, and 0.5 mg/ml) and blanks (containing only PBS). The collagen phantoms (Fig 2a) were prepared with collagen solutions and then freeze-dried under sterile conditions. The dried powder was resuspended in sterile 1mM HCl solution to form a high-purity, uniform collagen solution. After its preparation, the collagen solution was transferred to an imaging phantom and kept at low temperature (4°C). The collagen solution was then scanned using sPA imaging technology (wavelengths of 1070 to 1650 nm, 10 nm step size) to evaluate absorption behavior. The maximum fluence is 24 mJ/cm^2^ at 1070 nm, which is about 25% of the maximum permissible exposure (MPE) [65]. The CWRs were then calculated by the sPA unmixing method, and the range of detected CWRs was normalized to the highest concentration of the prepared collagen samples (1 mg/ml). The detected results are shown in Fig 2b, which indicated that the proposed sPA wavelength unmixing method was sensitive in its detection of the CWR in controlled collagen samples. Additionally, the concentration of collagen gels used in this study was below the collagen concentration in cervical tissue [15,66–68]. Therefore, the sPA measurement of collagen variations was anticipated to be even more sensitive in *in-vivo* human studies.

### CWR in tissue samples of the human cervix

We performed another study in human cervical samples to indicate the capability of the sPA method to detect the CWR. Wide-spectrum range sPA (from 1150 nm to 1650 nm) imaging was acquired, and the laser energies at different wavelengths were recorded to normalize the sPA signals with respect to energy variations, with the maximum fluence of 20 mJ/cm^2^ at 1150 nm. Although water is generally diffuse in soft tissue, the light beam acts locally. Hence, it produces the gradient required for generating PA signals. RNALater solution was also spectroscopically imaged in the same wavelength range to assure no interference from the background solution. Upon completion of the imaging studies, the tissue samples were washed with PBS, embedded into optimal cutting temperature compound, and underwent snap-freezing using liquid nitrogen, followed by storage at −80°C prior to histologic analyses. There were two types of stains applied to cervix samples: 1) Haemotoxylin and eosin (H&E) to depict edema (increased water content), in which water was visualized as void (uncolored) space [69], and 2) Sirius Red to semi-quantitatively determine the amount of collagen and non-collagen proteins [70]. By utilizing polarized light microscopic imaging, the organized concentric network of collagen can be visualized as a red/yellow rod-like structure.

Cervix samples from the hysterectomy groups were separated in those from women still in reproductive age (<41 years of age), and women at the end of the reproductive age (≥41 years old). We aimed to evaluate if changes in collagen and water content were related only to the effect of pregnancy and not by hormonal changes in the perimenopausal period.

## Results

### CWR in collagen phantoms

Fig 2 shows the sPA-CWR detection for the collagen phantoms. The collagen concentrations in the collagen phantoms ranged from 0% (PBS) to 1 mg/mL. The detection results were normalized to 1 mg/ml. The linear-fitted curve shows a high correlation (R^2^ = 0.9966) along with the collagen concentration increment. This result demonstrated that the sPA-CWR method is capable of detecting the collagen concentration in the collagen phantoms with high accuracy.

### CWR in samples of the human cervix

**Fig 3:**
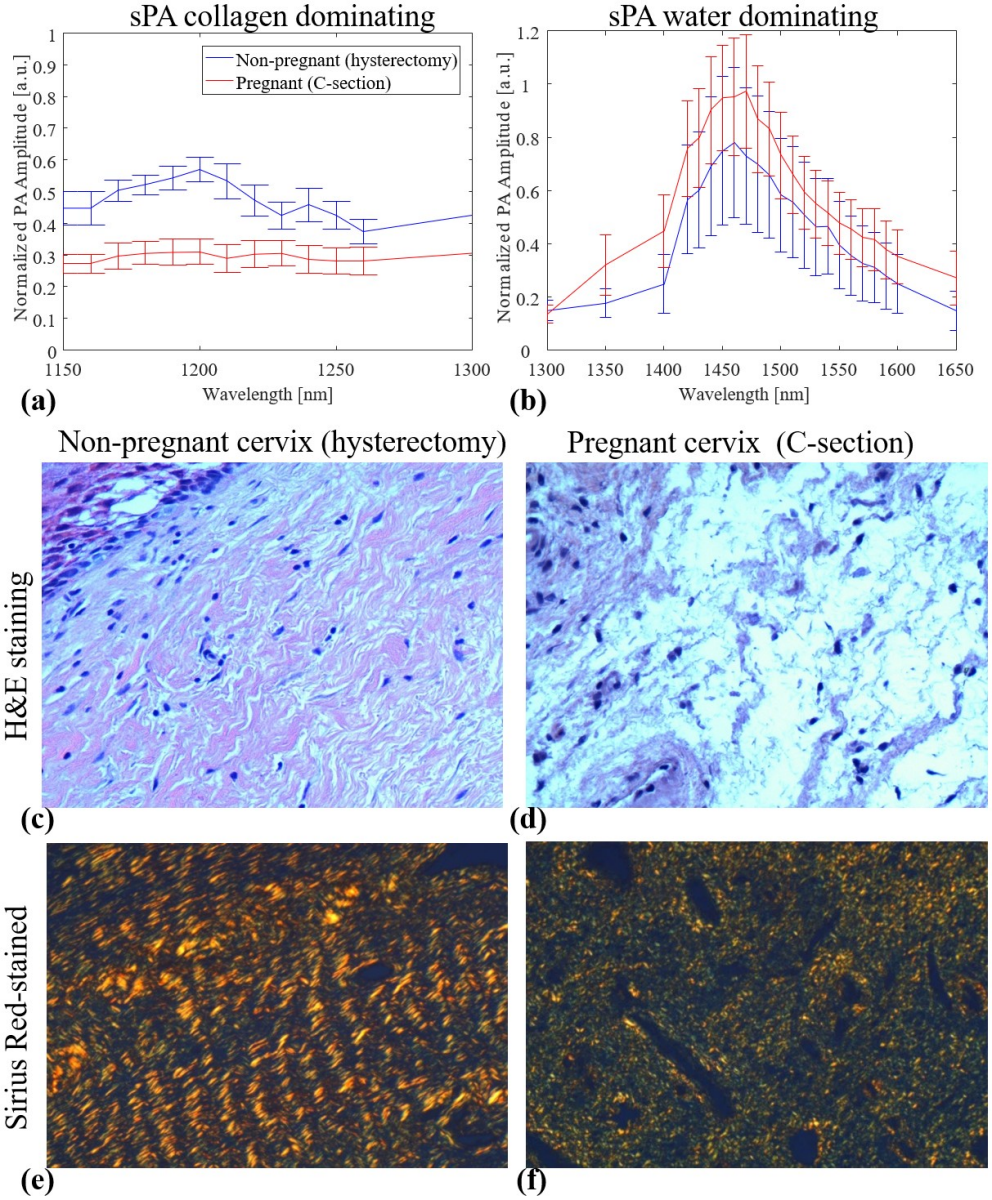
Averaged PA signal amplitudes and histological images of non-pregnant and pregnant women. (a, b) Spectroscopic photoacoustic (sPA) imaging signals of cervix samples obtained from pregnant and non-pregnant women (n = 8 for each group). The cervix samples from non-pregnant women showed higher PA signal amplitudes in the collagen absorption peak (around 1200 nm). By contrast, the cervix samples from pregnant women presented higher PA signal amplitudes in water absorption peak (around 1470 nm). (c, d) H&E-stained images indicated the loss of tissue composition (uncolored space) in non-pregnant cervix samples compare to pregnant cervix samples. (e, f) Sirius Red-stained images of non-pregnant and pregnant tissue visualized by polarized light microscope. The latter revealed less packed collagen fibers (red/yellow rod-like structure).

**Fig 4:**
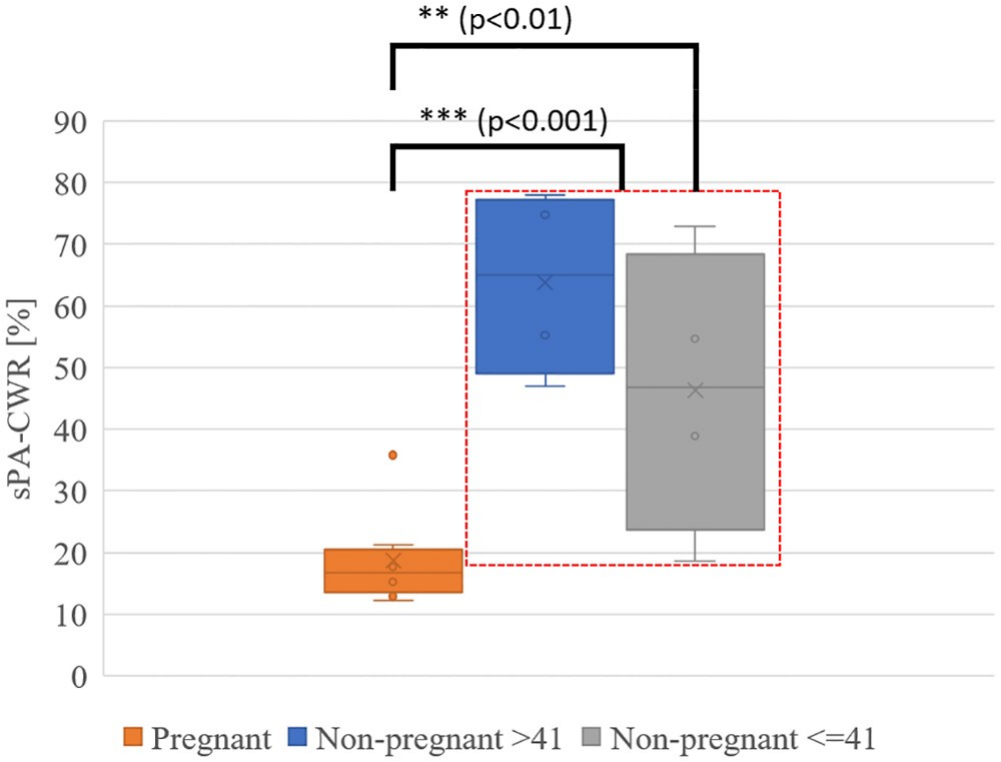
Spectroscopic photoacoustic (sPA) imaging estimated CWR in pregnant and non-pregnant ≤ 41 year-old and in non-pregnant > 41 year-old cervix samples. The analysis of all cervix samples from non-pregnant and pregnant women showed a significantly lower CWR in the pregnant cervix (at-term). A subgroup analysis comparing pregnant women and non-pregnant women age ≤ 41 years also indicated a significantly lower CWR in the cervix samples from pregnant women.

The age range for pregnant women was 21 to 36 years of age (median, 30 years), whereas it varied from 29 to 58 years (median, 42 years) for the hysterectomy group. In the hysterectomy group, there were four patients under 41 years of age and four patients over the age of 43 years. The demographic characteristics of all patients are detailed in Table 1. Fig 3 shows the averaged sPA amplitude changes across eight cervical tissue samples in each group along wavelengths, histological H&E staining, and Sirius Red-stained images of non-pregnant and pregnant women. From the detected PA signal amplitudes (Fig 3a and 3b), the non-pregnant cervical tissues had a clear peak from collagen absorptions in the collagen-dominating range (1150 to 1250 nm). By contrast, in the water-dominating range (1300 to 1650 nm), the cervical tissues from pregnant women had higher PA amplitude. H&E microscopic images (Fig 3c) of cervix samples taken from non-pregnant humans showed compact collagen bundles with minimal or no intervening edema. When examined with Sirius Red stain, it was evident that the collagen fibers form bundles with distinct fasciculation (Fig 3e). By contrast, the cervical samples from pregnant women showed loose collagen fibers with no discrete bundles and the presence of water (edema) separating the collagen fibers (Fig 3d). The absence of discrete collagen bundles, as well as the loss of the distinct fasciculation seen in samples from non-pregnant women, was evident (Fig 3f). We further quantified the CWR with the sPA wavelength unmixing method for pregnant (18.7%, SD 7.5%) and non-pregnant (55.0%, SD 20.3%) women and demonstrated a significant higher CWR in the non-pregnant group (two samples one-tailed t-test with equal variance, p = 0.00016). Subgroup analysis in pregnant vs. non-pregnant women <41 years of age also demonstrated a significant difference in the CWR between these two groups (pregnant 18.7%, SD 7.5% and non-pregnant ≤ 41 years, 46.3%, SD 23.1%; p = 0.0049) (Fig 4). The detected CWRs for each sample are shown in Table 1.

**Table 1:** Patient information and collagen-to-water ratio (CWR) [%] estimated by spectroscopic photoacoustic (sPA) imaging.

Sample No	Age (Years)	Ethnicity	Cervical Length [mm]	Gestational Age [Weeks]	BMI	sPA-CWR [%]
**Pregnant Cervical Samples**						
1	29	AA	38.5	39.4	19.6	18.5
2	28	AA	42.0	39.3	12.6	12.3
3	38	AA	28.0	39.0	16.5	15.8
4	28	AA	22.5	40.3	18.6	17.7
5	24	AA	23.7	39.6	39.0	35.8
6	36	AA	23.6	38.0	22.7	21.3
7	32	AA	29.0	39.7	15.9	15.2
8	21	AA	28.6	39.1	13.3	12.9
**Non-Pregnant Cervical Samples > 41 years of age**						
1	45	AA			22.2	55.3
2	50	AA			31.6	74.8
3	58	AA			27.7	77.9
4	43	AA			21.4	47.0
**Non-pregnant Cervical Samples ≤ 41 years of age**						
1	41	Other			30.0	18.6
2	29	AA			37.3	72.9
3	36	Other			32.9	38.9
4	37	AA			27.2	54.7

AA, African American; BMI, Body mass index; Other, Caucasian/Oriental/Hispanic.

## Discussion

The main finding of this study is that photoacoustic imaging showed significant differences in the CWR between cervical samples from pregnant and non-pregnant women. Cervical tissue from pregnant women at term showed a higher proportion of water and a lower proportion of collagen than samples from non-pregnant women. The proposed novel imaging spectroscopic photoacoustic estimation of collagen-to-water ratio (sPA-CWR) method is a non-invasive imaging modality that can provide important information on the cervical structure and its changes in the remodeling process during pregnancy and labor and after delivery.

The results of this study are in accordance with our previous observations in murine pregnancy, for which the collagen-and-water content changed during cervical remodeling, demonstrated by using PA imaging [46]. Combined US and PA techniques were used to evaluate cervical remodeling using *in-vitro* extracted collagen phantoms and *ex-vivo* murine cervical tissues collected at mid-pregnancy and term. The distinct sPA imaging (sPA patterns between collagen and water) delineated the cervical collagen network and cervical changes occurring during pregnancy. The sPA imaging accuracy to unmixing two spectroscopic separated absorbers had been determined as an error within 5% in our previous study [46].

The PA imaging findings were also supported by the histological analyses of the cervical tissue samples for pregnant and non-pregnant women. H&E staining images showed increased water content (more uncolored space) in pregnant women as compared to non-pregnant women. The polarized light microscope imaging with Sirius Red stain indicated that the non-pregnant group had a denser and more organized concentric network of collagen fibers than the pregnant group. These findings were also supported by previous literature reports that utilized optical imaging devices such as SHG and OCT in animal models [34,71–73].

The developed US/PA imaging system is capable of meeting the safety aspects required in a labor and delivery unit. The system provides enclosed laser illumination with fiber optics that directs the beam to the cervix. The laser energy can be controlled in real-time with a power meter during the operation. The fluence required for imaging the cervix’s stroma layer is 24 mJ/cm^2^ at 1070 nm, which is less than 25% of the MPE defined by American National Standard Institute (ANSI) guideline for safe use of lasers [65]. Additionally, the TVUS/PA safety has been reported for *in-vivo* imaging of human ovarian cancer (requiring greater penetration depth than our application) [74,75], and its safety is also regarded in an ongoing clinical trial [76].

### Potential clinical application

This study demonstrates, for the first time, the use of PA imaging to evaluate the characteristics of the cervix at term by using human cervical tissue. This emerging modality may serve as a promising ancillary tool to provide real-time, high-resolution molecular images by using non-ionizing radiation and the added advantage of compatibility with an ultrasound imaging system [77]. The acquisition of valuable information of cervical changes occurring at the molecular level during pregnancy by means of a non-invasive approach may be used to increase the predictive accuracy of conventional transvaginal sonography if combined with the PA imaging modality. Photoacoustic imaging could also be used to assess patient response to therapy, which can help to individualize the clinical management with progesterone, cerclage, or pessary in women at risk for spontaneous preterm birth [78–87]. This bimodal imaging method can potentially acquire data for tissue elasticity, blood perfusion, and tissue oxygenation. The single device is capable of measuring anatomical, biomechanical, molecular, and functional characteristics of the cervix and has the potential of optimizing the time for induction of labor.

### Strengths and weaknesses

**Fig 5:**
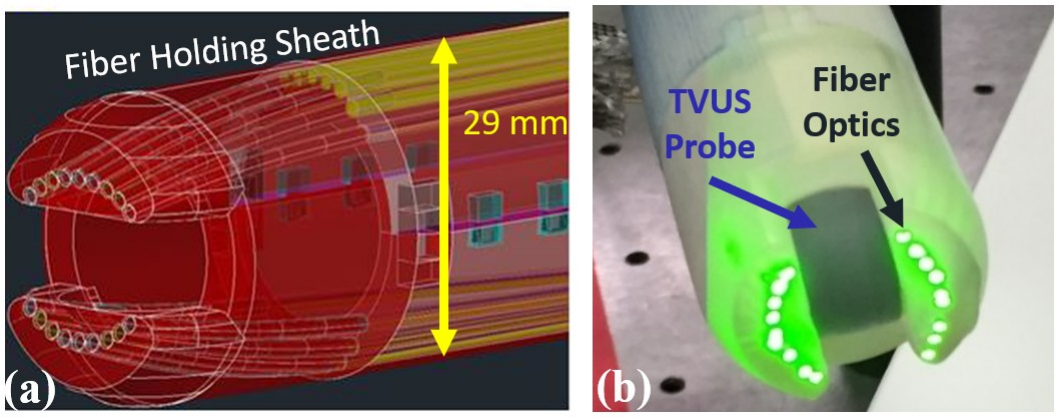
Transvaginal ultrasound and photoacoustic imaging system for future *in-vivo* studies. (a) Computer-assisted 3D design of the fiber-optical delivery system around a clinical transvaginal ultrasound transducer. (b) A photograph of the assembled combined photoacoustic (PA) and transvaginal ultrasound imaging transducer. This prototype can be a point-of-care device for the future clinical diagnoses by characterizing cervix status. The diameter of this prototype is 29 mm, which satisfies clinical requirements.

Among its strengths, the proposed imaging system is more advanced than standard TVUS and sonographic evaluation for cervical length; it can supplement the existing, widely used TVUS; and it has a high safety standard, making it reliable to use for pregnant women [88]. We anticipate that the sPA measurements can be performed in 3–5 minutes. The additional requirement of sPA imaging can be easily combined with the current ultrasound system, thus providing a point-of-care diagnostic tool. The whole system, including the laser, control, and ultrasound unit, can be easily fitted in a portable cart of similar size to a clinical ultrasound machine. The device holds the potential for a clinically acceptable routine examination capable of identifying cervical changes during pregnancy and throughout labor induction in a labor and delivery unit. Fig 5 shows the design and physical dimension of a developed prototype with current TVUS transducers.

We noted certain limitations of the study and the PA imaging. The statistical results obtained in this study were limited given the small sample size. Although the sPA-CWRs were estimated *in-vitro*, it is still feasible to detect cervical changes leading to spontaneous preterm birth at a larger scale in the *in-vivo* study. The lack of Bishop scores associated with the pregnant cervices may lead to the loss of valuable information. The estimation of sPA-CWR of the whole cervix in PA imaging cannot be evaluated at one scanning. Data should be obtained from different cervical areas to create a global PA cervical score. A higher frequency linear US probe was used to image samples of about five cubic millimeters. By contrast, a TVUS probe set at lower frequency and bandwidth may offer a different sensitivity. The sensitivity may also vary during *in-vivo* studies. Future studies will focus on patients presenting a torn or insufficient cervix who undergo labor; however, the cervices of cesarean deliveries may not be ripened at the time of the study. Future studies are also required to determine the cost-effectiveness and long-term effects of this modality.

## Conclusions

This research demonstrated the abilities of a combined US and sPA imaging system and method that measures the cervical tissue optical properties in the near-infrared range. From the imaging results of 16 samples, the PA imaging of CWR can detect variations in the collagen organization in the human cervix between the non-pregnant and cesarean delivery groups. Significant differences were indicated in the CWR of cervical samples obtained from pregnant and non-pregnant women. In addition, we observed detectable spectral changes, probably reflecting the cervical collagen network organization. Moreover, we compared the sPA results with histological tissue analysis of the human cervix and confirmed the capability of the bimodal system to evaluate the process of cervical remodeling accurately. The proposed imaging system will pave the way toward achieving reliable screening/diagnosis with higher sensitivity and specificity to detect early signs of cervical insufficiency and as a potential method to predict preterm birth.

## Supporting information

S1 File (7Z)

## References

[1] Nott JP, Bonney EA, Pickering JD, Simpson NA. The structure and function of the cervix during pregnancy. Translational Research in Anatomy. 2016;2:1–7.

[2] Timmons B, Akins M, Mahendroo M. Cervical remodeling during pregnancy and parturition. Trends in Endocrinology & Metabolism. 2010;21(6):353–61. 10.1016/j.tem.2010.01.011 20172738 PMC2880223

[3] Word RA, Li X-H, Hnat M, Carrick K, editors. Dynamics of cervical remodeling during pregnancy and parturition: mechanisms and current concepts. Seminars in reproductive medicine; 2007: Copyright© 2007 by Thieme Publishers, Inc., 333 Seventh Avenue, New York, NY 10001, USA. 10.1055/s-2006-956777 17205425

[4] Ludmir J, Sehdev HM. Anatomy and physiology of the uterine cervix. Clin Obstet Gynecol. 2000;43(3):433–9. Epub 2000/08/19. 10.1097/00003081-200009000-00003 . 10949747

[5] Gonzalez JM, Dong Z, Romero R, Girardi G. Cervical remodeling/ripening at term and preterm delivery: the same mechanism initiated by different mediators and different effector cells. PLoS One. 2011;6(11):e26877. Epub 2011/11/11. 10.1371/journal.pone.0026877 . 22073213 PMC3206857

[6] Vink JY, Qin S, Brock CO, Zork NM, Feltovich HM, Chen X, et al. A new paradigm for the role of smooth muscle cells in the human cervix. American journal of obstetrics and gynecology. 2016;215(4):478.e1–e11. 10.1016/j.ajog.2016.04.053 27166013

[7] Muñoz-de-Toro M, Varayoud J, Ramos JG, Rodríguez HA, Luque EH. Collagen remodeling during cervical ripening is a key event for successful vaginal delivery. Braz J Morphol Sci. 2003;20:75–84.

[8] Nallasamy S, Yoshida K, Akins M, Myers K, Iozzo R, Mahendroo M. Steroid hormones are key modulators of tissue mechanical function via regulation of collagen and elastic fibers. Endocrinology. 2017;158(4):950–62. 10.1210/en.2016-1930 28204185 PMC5460796

[9] Zork NM, Myers KM, Yoshida K, Cremers S, Jiang H, Ananth CV, et al. A systematic evaluation of collagen cross-links in the human cervix. American journal of obstetrics and gynecology. 2015;212(3):321.e1–e8. 10.1016/j.ajog.2014.09.036 25281365 PMC4346506

[10] Yoshida K, Jiang H, Kim M, Vink J, Cremers S, Paik D, et al. Quantitative evaluation of collagen crosslinks and corresponding tensile mechanical properties in mouse cervical tissue during normal pregnancy. PloS one. 2014;9(11):e112391. 10.1371/journal.pone.0112391 25397407 PMC4232411

[11] Nallasamy S, Akins M, Tetreault B, Luby-Phelps K, Mahendroo M. Distinct reorganization of collagen architecture in lipopolysaccharide-mediated premature cervical remodeling. Biology of reproduction. 2017;98(1):63–74. 10.1093/biolre/iox155 PMC5803761 29161343

[12] Akins ML, Luby-Phelps K, Bank RA, Mahendroo M. Cervical softening during pregnancy: regulated changes in collagen cross-linking and composition of matricellular proteins in the mouse. Biology of reproduction. 2011;84(5):1053–62. 10.1095/biolreprod.110.089599 21248285 PMC3080426

[13] Cunningham F, Leveno K, Bloom S, Spong CY, Dashe J. Williams obstetrics, 24e: Mcgraw-hill; 2014.

[14] Uldbjerg N, Ekman G, Malmström A, Olsson K, Ulmsten U. Ripening of the human uterine cervix related to changes in collagen, glycosaminoglycans, and collagenolytic activity. American journal of obstetrics and gynecology. 1983;147(6):662–6. 10.1016/0002-9378(83)90446-5 6638110

[15] Uldbjerg N, Ekman G, Malmström A, Olsson K, Ulmsten U. Ripening of the human uterine cervix related to changes in collagen, glycosaminoglycans, and collagenolytic activity. American Journal of Obstetrics & Gynecology. 1983;147(6):662–6. 6638110 10.1016/0002-9378(83)90446-5

[16] Timmons B, Akins M, Mahendroo M. Cervical remodeling during pregnancy and parturition. Trends in endocrinology and metabolism: TEM. 2010;21(6):353–61. Epub 2010/02/23. 10.1016/j.tem.2010.01.011 . 20172738 PMC2880223

[17] Word RA, Li XH, Hnat M, Carrick K. Dynamics of cervical remodeling during pregnancy and parturition: mechanisms and current concepts. Seminars in reproductive medicine. 2007;25(1):69–79. Epub 2007/01/06. 10.1055/s-2006-956777 . 17205425

[18] Akins ML, Luby-Phelps K, Bank RA, Mahendroo M. Cervical softening during pregnancy: regulated changes in collagen cross-linking and composition of matricellular proteins in the mouse. Biology of reproduction. 2011;84(5):1053–62. Epub 2011/01/21. 10.1095/biolreprod.110.089599 . 21248285 PMC3080426

[19] Feltovich H, Nam K, Hall TJ. Quantitative ultrasound assessment of cervical microstructure. Ultrasonic imaging. 2010;32(3):131–42. Epub 2010/08/20. 10.1177/016173461003200302 . 20718243

[20] Feltovich H, Hall TJ, Berghella V. Beyond cervical length: emerging technologies for assessing the pregnant cervix. American journal of obstetrics and gynecology. 2012;207(5):345–54. Epub 2012/06/22. 10.1016/j.ajog.2012.05.015 . 22717270 PMC3458165

[21] Taipale P, Hiilesmaa V. Sonographic measurement of uterine cervix at 18–22 weeks’ gestation and the risk of preterm delivery. Obstetrics & Gynecology. 1998;92(6):902–7. 9840546 10.1016/s0029-7844(98)00346-9

[22] Mella MT, Berghella V, editors. Prediction of preterm birth: cervical sonography. Seminars in perinatology; 2009: Elsevier. 10.1053/j.semperi.2009.06.007 19796729

[23] O’Hara S, Zelesco M, Sun Z. Cervical length for predicting preterm birth and a comparison of ultrasonic measurement techniques. Australasian journal of ultrasound in medicine. 2013;16(3):124–34. 10.1002/j.2205-0140.2013.tb00100.x 28191186 PMC5029998

[24] Hassan SS, Romero R, Vidyadhari D, Fusey S, Baxter JK, Khandelwal M, et al. Vaginal progesterone reduces the rate of preterm birth in women with a sonographic short cervix: a multicenter, randomized, double-blind, placebo-controlled trial. Ultrasound in obstetrics & gynecology: the official journal of the International Society of Ultrasound in Obstetrics and Gynecology. 2011;38(1):18–31. Epub 2011/04/08. 10.1002/uog.9017 . 21472815 PMC3482512

[25] Mahendroo M. Cervical remodeling in term and preterm birth: insights from an animal model. Reproduction. 2012;143(4):429–38. 10.1530/REP-11-0466 22344465

[26] Tan P, Vallikkannu N, Suguna S, Quek K, Hassan J. Transvaginal sonographic measurement of cervical length vs. Bishop score in labor induction at term: tolerability and prediction of Cesarean delivery. Ultrasound in Obstetrics and Gynecology: The Official Journal of the International Society of Ultrasound in Obstetrics and Gynecology. 2007;29(5):568–73. 10.1002/uog.4018 17444553

[27] Ware V, Raynor BD. Transvaginal ultrasonographic cervical measurement as a predictor of successful labor induction. American journal of obstetrics and gynecology. 2000;182(5):1030–2. 10.1067/mob.2000.105399 10819818

[28] Hwang HS, Sohn IS, Kwon HS. Imaging analysis of cervical elastography for prediction of successful induction of labor at term. Journal of Ultrasound in Medicine. 2013;32(6):937–46. 10.7863/ultra.32.6.937 23716514

[29] Swiatkowska-Freund M, Preis K. Elastography of the uterine cervix: implications for success of induction of labor. Ultrasound in Obstetrics & Gynecology. 2011;38(1):52–6. 10.1002/uog.9021 21484905

[30] O’Brien CM, Herington JL, Brown N, Pence IJ, Paria BC, Slaughter JC, et al. In vivo Raman spectral analysis of impaired cervical remodeling in a mouse model of delayed parturition. Sci Rep. 2017;7(1):6835. Epub 2017/07/30. 10.1038/s41598-017-07047-5 . 28754971 PMC5533720

[31] Maul H, Saade G, Garfield RE. Prediction of term and preterm parturition and treatment monitoring by measurement of cervical cross-linked collagen using light-induced fluorescence. Acta obstetricia et gynecologica Scandinavica. 2005;84(6):534–6. Epub 2005/05/20. 10.1111/j.0001-6349.2005.00806.x . 15901259

[32] Zhang Y, Akins ML, Murari K, Xi J, Li MJ, Luby-Phelps K, et al. A compact fiber-optic SHG scanning endomicroscope and its application to visualize cervical remodeling during pregnancy. Proceedings of the National Academy of Sciences of the United States of America. 2012;109(32):12878–83. Epub 2012/07/25. 10.1073/pnas.1121495109 . 22826263 PMC3420182

[33] Baños A, Wolf M, Grawe C, Stahel M, Haensse D, Fink D, et al. Frequency domain near‐infrared spectroscopy of the uterine cervix during cervical ripening. Lasers in Surgery and Medicine: The Official Journal of the American Society for Laser Medicine and Surgery. 2007;39(8):641–6. 10.1002/lsm.20542 17886282

[34] Yao W, Gan Y, Myers KM, Vink JY, Wapner RJ, Hendon CP. Collagen Fiber Orientation and Dispersion in the Upper Cervix of Non-Pregnant and Pregnant Women. PLoS One. 2016;11(11):e0166709. Epub 2016/11/30. 10.1371/journal.pone.0166709 . 27898677 PMC5127549

[35] Gan Y, Yao W, Myers KM, Vink JY, Wapner RJ, Hendon CP. Analyzing three-dimensional ultrastructure of human cervical tissue using optical coherence tomography. Biomedical optics express. 2015;6(4):1090–108. Epub 2015/04/25. 10.1364/BOE.6.001090 . 25908997 PMC4399652

[36] Wang LV, Hu S. Photoacoustic tomography: in vivo imaging from organelles to organs. Science. 2012;335(6075):1458–62. Epub 2012/03/24. 10.1126/science.1216210 . 22442475 PMC3322413

[37] Cox B, Laufer JG, Arridge SR, Beard PC. Quantitative spectroscopic photoacoustic imaging: a review. Journal of biomedical optics. 2012;17(6):061202. Epub 2012/06/28. 10.1117/1.JBO.17.6.061202 . 22734732

[38] Wang P, Rajian JR, Cheng JX. Spectroscopic Imaging of Deep Tissue through Photoacoustic Detection of Molecular Vibration. The journal of physical chemistry letters. 2013;4(13):2177–85. Epub 2013/09/28. 10.1021/jz400559a . 24073304 PMC3780401

[39] Yang JM, Favazza C, Chen R, Yao J, Cai X, Maslov K, et al. Simultaneous functional photoacoustic and ultrasonic endoscopy of internal organs in vivo. Nature medicine. 2012;18(8):1297–302. Epub 2012/07/17. 10.1038/nm.2823 . 22797808 PMC3885361

[40] Li C, Yang JM, Chen R, Yeh CH, Zhu L, Maslov K, et al. Urogenital photoacoustic endoscope. Optics letters. 2014;39(6):1473–6. Epub 2014/04/03. 10.1364/ol.39.001473 . 24690816 PMC4009352

[41] Wang X, Xie X, Ku G, Wang LV, Stoica G. Non-invasive imaging of hemoglobin concentration and oxygenation in the rat brain using high-resolution photoacoustic tomography. Journal of biomedical optics. 2006;11(2):024015. 10.1117/1.2192804 16674205

[42] MehrmohammadiM, Hernandez-AndradeE, GelovaniJG, HassanSS, YanY. Ultrasound and photoacoustic systems and methods for fetal brain assessment during delivery. Google Patents; 2018.

[43] Tian C, Xie Z, Fabiilli ML, Wang X. Imaging and sensing based on dual-pulse nonlinear photoacoustic contrast: a preliminary study on fatty liver. Optics letters. 2015;40(10):2253–6. 10.1364/OL.40.002253 26393712 PMC4581454

[44] Yan Y, Basij M, Wang Z, Siddiqui A, Dong J, Alijabbari N, et al., editors. Multi-parametric acoustic imaging of cervix for more accurate detection of patients at risk of preterm birth. 2018 IEEE International Ultrasonics Symposium (IUS); 2018: IEEE.

[45] Yan Y, Dong J, Siddiqu AA, Majalikar Y, Basij M, Hernandez-Andrade E, et al., editors. Ultrasound, elasticity, and photoacoustic imaging of cervix: towards a more accurate prediction of preterm delivery (Conference Presentation). Medical Imaging 2018: Ultrasonic Imaging and Tomography; 2018: International Society for Optics and Photonics.

[46] Yan Y, Gomez-Lopez N, Basij M, Shahvari AV, Vadillo-Ortega F, Hernandez-Andrade E, et al. Photoacoustic imaging of the uterine cervix to assess collagen and water content changes in murine pregnancy. Biomedical optics express. 2019;10(9):4643–55. 10.1364/BOE.10.004643 31565515 PMC6757472

[47] Basij M, Yan Y, Alshahrani SS, Helmi H, Burton TK, Burmeister JW, et al. Miniaturized phased-array ultrasound and photoacoustic endoscopic imaging system. Photoacoustics. 2019;15:100139. 10.1016/j.pacs.2019.100139 31388487 PMC6677929

[48] Qu Y, Hu P, Shi J, Maslov K, Zhao P, Li C, et al. In vivo characterization of connective tissue remodeling using infrared photoacoustic spectra. Journal of biomedical optics. 2018;23(12):121621. 10.1117/1.JBO.23.12.121621 PMC6318810 30520275

[49] Dumani DS, Sun I-C, Emelianov SY. Ultrasound-guided immunofunctional photoacoustic imaging for diagnosis of lymph node metastases. Nanoscale. 2019;11(24):11649–59. 10.1039/c9nr02920f 31173038 PMC6586492

[50] Allen TJ, Hall A, Dhillon AP, Owen JS, Beard PC. Spectroscopic photoacoustic imaging of lipid-rich plaques in the human aorta in the 740 to 1400 nm wavelength range. Journal of biomedical optics. 2012;17(6):061209. Epub 2012/06/28. 10.1117/1.JBO.17.6.061209 . 22734739

[51] Alshahrani S, Yan Y, Avrutsky I, Malyarenko E, Anastasio M, Mehrmohammadi M, editors. An advanced photoacoustic tomography system based on a ring geometry design. Medical Imaging 2018: Ultrasonic Imaging and Tomography; 2018: International Society for Optics and Photonics.

[52] Oh J-T, Li M-L, Zhang HF, Maslov K, Wang LV. Three-dimensional imaging of skin melanoma in vivo by dual-wavelength photoacoustic microscopy. Journal of biomedical optics. 2006;11(3):034032. 10.1117/1.2210907 16822081

[53] Basij M, Yan Y, Alshahrani S, Winer I, Burmeister J, Dominello M, et al., editors. Development of an Ultrasound and Photoacoustic Endoscopy System for Imaging of Gynecological Disorders. 2018 IEEE International Ultrasonics Symposium (IUS); 2018: IEEE.

[54] Mallidi S, Luke GP, Emelianov S. Photoacoustic imaging in cancer detection, diagnosis, and treatment guidance. Trends in biotechnology. 2011;29(5):213–21. Epub 2011/02/18. 10.1016/j.tibtech.2011.01.006 . 21324541 PMC3080445

[55] Mehrmohammadi M, Yoon SJ, Yeager D, Emelianov SY. Photoacoustic Imaging for Cancer Detection and Staging. Current molecular imaging. 2013;2(1):89–105. Epub 2013/09/14. 10.2174/2211555211302010010 . 24032095 PMC3769095

[56] Alshahrani S, Yan Y, Avrutsky I, Anastasio M, Malyarenko E, Duric N, et al., editors. Design and development of a full-ring ultrasound and photoacoustic tomography system for breast cancer imaging. 2017 IEEE International Ultrasonics Symposium (IUS); 2017: IEEE.

[57] Gargiulo S, Albanese S, Mancini M. State-of-the-Art preclinical photoacoustic imaging in oncology: recent advances in cancer theranostics. Contrast media & molecular imaging. 2019;2019. 10.1155/2019/5080267 31182936 PMC6515147

[58] Qu Y, Hu P, Shi J, Maslov K, Zhao P, Li C, et al. In vivo characterization of connective tissue remodeling using infrared photoacoustic spectra. Journal of biomedical optics. 2018;23(12):1–6. Epub 2018/12/07. 10.1117/1.JBO.23.12.121621 PMC6318810 30520275

[59] Yan Y, Kondle S, Mehrmohammadi M. Comparing Different Fiber Guided Light Delivery Strategies in an Endocavity Photoacoustic Imaging System: A Monte-Carlo Simulation Study. J Biomed Res Prac. 2019;3(1):100015.

[60] Park S, Karpiouk AB, Aglyamov SR, Emelianov SY. Adaptive beamforming for photoacoustic imaging. Optics letters. 2008;33(12):1291–3. Epub 2008/06/17. 10.1364/ol.33.001291 . 18552935 PMC2713818

[61] Sekar SKV, Bargigia I, Dalla Mora A, Taroni P, Ruggeri A, Tosi A, et al. Diffuse optical characterization of collagen absorption from 500 to 1700 nm. Journal of biomedical optics. 2017;22(1):015006. 10.1117/1.JBO.22.1.015006 28138693

[62] Sordillo DC, Sordillo LA, Sordillo PP, Shi L, Alfano RR. Short wavelength infrared optical windows for evaluation of benign and malignant tissues. Journal of biomedical optics. 2017;22(4):045002. 10.1117/1.JBO.22.4.045002 28384701

[63] Nachabé R, Evers DJ, Hendriks BH, Lucassen GW, van der Voort M, Rutgers EJ, et al. Diagnosis of breast cancer using diffuse optical spectroscopy from 500 to 1600 nm: comparison of classification methods. Journal of biomedical optics. 2011;16(8):087010–12. 10.1117/1.3611010 21895337

[64] Dumani D, Sun I-C, Emelianov S, editors. In vivo photoacoustic detection of lymph node metastasis using glycol-chitosan-coated gold nanoparticles. Ultrasonics Symposium (IUS), 2017 IEEE International; 2017: IEEE.

[65] Institute ANS. American national standard for safe use of lasers: Laser Institute of America; 2014.

[66] Oxlund BS, Ørtoft G, Brüel A, Danielsen CC, Bor P, Oxlund H, et al. Collagen concentration and biomechanical properties of samples from the lower uterine cervix in relation to age and parity in non-pregnant women. Reproductive Biology and Endocrinology. 2010;8(1):82. 10.1186/1477-7827-8-82 20604933 PMC2907383

[67] Kleissl H, Van Der Rest M, Naftolin F, Glorieux FH, De Leon A. Collagen changes in the human uterine cervix at parturition. American journal of obstetrics and gynecology. 1978;130(7):748–53. 10.1016/0002-9378(78)90003-0 637097

[68] Ito A, Kitamura K, Mori Y, Hirakawa S. The change in solubility of type I collagen in human uterine cervix in pregnancy at term. Biochemical medicine. 1979;21(3):262–70. 10.1016/0006-2944(79)90081-4 496919

[69] Fischer AH, Jacobson KA, Rose J, Zeller R. Hematoxylin and eosin staining of tissue and cell sections. Cold spring harbor protocols. 2008;2008(5):. 10.1101/pdb.prot4986 21356829

[70] López-De León A, Rojkind M. A simple micromethod for collagen and total protein determination in formalin-fixed paraffin-embedded sections. Journal of Histochemistry & Cytochemistry. 1985;33(8):737–43. 10.1177/33.8.2410480 2410480

[71] Narice BF, Green NH, MacNeil S, Anumba D. Second Harmonic Generation microscopy reveals collagen fibres are more organised in the cervix of postmenopausal women. Reproductive Biology and Endocrinology. 2016;14(1):1–8. 10.1186/s12958-016-0204-7 27769268 PMC5073459

[72] Akins ML, Luby-Phelps K, Mahendroo M. Second harmonic generation imaging as a potential tool for staging pregnancy and predicting preterm birth. Journal of biomedical optics. 2010;15(2):026020. 10.1117/1.3381184 20459265 PMC2874049

[73] Myers K, Socrate S, Tzeranis D, House M. Changes in the biochemical constituents and morphologic appearance of the human cervical stroma during pregnancy. European Journal of Obstetrics & Gynecology and Reproductive Biology. 2009;144:S82–S9. 10.1016/j.ejogrb.2009.02.008 19303693

[74] Mostafa A, Nandy S, Amidi E, Zhu Q, editors. Dual-mode photoacoustic and ultrasound system for real-time in-vivo ovarian cancer imaging. Photons Plus Ultrasound: Imaging and Sensing 2018; 2018: International Society for Optics and Photonics.

[75] Nandy S, Mostafa A, Zhu Q, editors. In vivo imaging of human ovarian cancer using co-registered ultrasound and photoacoustic tomography (Conference Presentation). Photons Plus Ultrasound: Imaging and Sensing 2018; 2018: International Society for Optics and Photonics.

[76] St Louis D, Romero R, Plazyo O, Arenas-Hernandez M, Panaitescu B, Xu Y, et al. Invariant NKT Cell Activation Induces Late Preterm Birth That Is Attenuated by Rosiglitazone. Journal of immunology (Baltimore, Md: 1950). 2016;196(3):1044–59. Epub 2016/01/08. 10.4049/jimmunol.1501962 . 26740111 PMC4724534

[77] Zackrisson S, Van De Ven S, Gambhir S. Light in and sound out: emerging translational strategies for photoacoustic imaging. Cancer research. 2014;74(4):979–1004. 10.1158/0008-5472.CAN-13-2387 24514041 PMC3944207

[78] Berghella V, Ciardulli A, Rust OA, To M, Otsuki K, Althuisius S, et al. Cerclage for sonographic short cervix in singleton gestations without prior spontaneous preterm birth: systematic review and meta-analysis of randomized controlled trials using individual patient-level data. Ultrasound in obstetrics & gynecology: the official journal of the International Society of Ultrasound in Obstetrics and Gynecology. 2017;50(5):569–77. Epub 2017/03/16. 10.1002/uog.17457 . 28295722

[79] Szychowski JM, Owen J, Hankins G, Iams JD, Sheffield JS, Perez-Delboy A, et al. Can the optimal cervical length for placing ultrasound-indicated cerclage be identified? Ultrasound in obstetrics & gynecology: the official journal of the International Society of Ultrasound in Obstetrics and Gynecology. 2016;48(1):43–7. Epub 2015/08/19. 10.1002/uog.15674 . 26277877 PMC6918708

[80] Suhag A, Saccone G, Bisulli M, Seligman N, Berghella V. Trends in cerclage use. Acta obstetricia et gynecologica Scandinavica. 2015;94(11):1188–94. Epub 2015/08/08. 10.1111/aogs.12725 . 26249133

[81] Suhag A, Berghella V. Cervical cerclage. Clin Obstet Gynecol. 2014;57(3):557–67. Epub 2014/07/01. 10.1097/GRF.0000000000000044 . 24979354

[82] Dorairajan G, Pegu B. Pessary Compared With Vaginal Progesterone for the Prevention of Preterm Birth in Women With Twin Pregnancies and Cervical Length Less Than 38 mm: A Randomized Controlled Trial. Obstetrics and gynecology. 2019;133(6):1283. Epub 2019/05/29. 10.1097/AOG.0000000000003297 . 31135748

[83] Stafford IA, Garite TJ, Maurel K, Combs CA, Heyborne K, Porreco R, et al. Cervical Pessary versus Expectant Management for the Prevention of Delivery Prior to 36 Weeks in Women with Placenta Previa: A Randomized Controlled Trial. AJP reports. 2019;9(2):e160–e6. Epub 2019/05/03. 10.1055/s-0039-1687871 . 31044098 PMC6491366

[84] Nicolaides KH, Syngelaki A, Poon LC, Picciarelli G, Tul N, Zamprakou A, et al. A Randomized Trial of a Cervical Pessary to Prevent Preterm Singleton Birth. The New England journal of medicine. 2016;374(11):1044–52. Epub 2016/03/18. 10.1056/NEJMoa1511014 . 26981934

[85] Alfirevic Z, Owen J, Carreras Moratonas E, Sharp AN, Szychowski JM, Goya M. Vaginal progesterone, cerclage or cervical pessary for preventing preterm birth in asymptomatic singleton pregnant women with a history of preterm birth and a sonographic short cervix. Ultrasound in obstetrics & gynecology: the official journal of the International Society of Ultrasound in Obstetrics and Gynecology. 2013;41(2):146–51. Epub 2012/09/20. 10.1002/uog.12300 . 22991337

[86] Stricker N, Timmesfeld N, Kyvernitakis I, Goerges J, Arabin B. Vaginal progesterone combined with cervical pessary: A chance for pregnancies at risk for preterm birth? American journal of obstetrics and gynecology. 2016;214(6):739.e1–e10. Epub 2015/12/23. 10.1016/j.ajog.2015.12.007 . 26692180

[87] Ludmir J. Cervical pessary reduces spontaneous delivery before 34 weeks and adverse outcomes in pregnant women with a short cervix. Evidence-based medicine. 2013;18(3):107–8. Epub 2012/08/31. 10.1136/eb-2012-100864 . 22933547

[88] Standard A. Z136. 1. American national standard for the safe use of lasers. American National Standards Institute. Inc, New York. 1993.

